# Posterior segment findings by spectral-domain optical coherence tomography and clinical associations in active toxoplasmic retinochoroiditis

**DOI:** 10.1038/s41598-022-05070-9

**Published:** 2022-01-21

**Authors:** Genevieve F. Oliver, Lisia Barros Ferreira, Barbara R. Vieira, Sigrid Arruda, Michelle Araújo, Jillian M. Carr, Justine R. Smith, João M. Furtado

**Affiliations:** 1 Flinders University College of Medicine and Public Health, Adelaide, Australia; 2 Division of Ophthalmology, Ribeirão Preto Medical School, University of São Paulo, Avenida Bandeirantes 3900, Ribeirão Preto, São Paulo 14049-900 Brazil

**Keywords:** Eye diseases, Retinal diseases

## Abstract

Toxoplasmic retinochoroiditis is a common, potentially blinding parasitic infection. We sought to define the spectrum and frequency of signs of active toxoplasmic retinochoroiditis by spectral domain optical coherence tomography (SD-OCT), and to identify clinical associations. Ninety eyes of 90 individuals presenting consecutively to a tertiary referral uveitis service with active toxoplasmic retinochoroiditis and gradable SD-OCT scans were evaluated prospectively. SD-OCT features were collated, and associations with lesion location, primary versus recurrent episode, serological status, human immunodeficiency virus infection and best-corrected Snellen visual acuity were explored. Active toxoplasmic retinochoroiditis presented with thickened (65%) and hyperreflective (61%) retina, choroidal thickening (55%) and hyporeflectivity (61%), hyperreflective vitreous dots (80%) and deposits (36%), and posterior hyaloid thickening (35%) on SD-OCT. Most signs occurred with similar frequency across clinical groups. Retinal hyporeflectivity (17%) was significantly associated with a visual acuity of 20/200 or worse at resolution. Our observations demonstrate that active toxoplasmic retinochoroiditis has diverse SD-OCT signs and that none are universally present. Retinal hyporeflectivity—suggesting liquefactive necrosis—predicts poor visual outcome.

## Introduction

Toxoplasmic retinochoroiditis (TRC) is the most common disease caused by an infection with the parasite, *Toxoplasma gondii*^1^. There is striking variation in global prevalence of this disease. In the United States, TRC occurs in an estimated 21,000 individuals annually^2^. In South America, the infection is at least 10-times more common than in the United States^3^. Evaluation of donor eyes in eye banks across Brazil has identified TRC in 15%^4^. The diagnosis of active TRC is straightforward in cases with a “satellite lesion” of retinochoroiditis adjacent to a pigmented chorioretinal scar^5^. When there is no scar, however, the diagnosis is less obvious. In a study of 154 individuals with TRC, 28% presented with their first ocular infection and had no scars in either eye^6^. Serious visual sequelae result from TRC: the same study documented blindness in at least one eye in 24% of participants^6^.

Spectral domain optical coherence tomography (SD-OCT) is a well-established modality for imaging the structure of the posterior eye, with resolution of approximately 5 µm. A limited number of studies have used SD-OCT to identify signs of TRC. In the largest cohort study to date, 24 eyes of 24 patients with active TRC were examined, and common SD-OCT signs included posterior hyaloid thickening and retinal hyperreflectivity, thickening and disorganisation^7^. Smaller studies have included both active and healed lesions, and TRC-associated macular oedema and choroidal thickening^8–13^. Spectral domain OCT signs that indicate prognosis have not been reported.

We sought to identify signs of TRC on SD-OCT, to define frequency and determine differences between clinical subgroups (i.e. posterior pole vs. peripheral lesions, or primary vs. recurrent, or based on *T. gondii* and HIV serology results) and to identify signs that predicted visual outcome. We used SD-OCT to examine patients with TRC who presented consecutively over a 34-month period to a uveitis practice in an area of Brazil with high prevalence of toxoplasmosis.

## Methods

### Patient selection

From 14 January 2015 to 16 November 2017, consecutive patients, diagnosed with TRC and aged over 12 years, were prospectively recruited at the Uveitis Clinic at Ribeirão Preto General Hospital. This tertiary referral clinic serves a population of approximately 1.7 million people in the state of São Paulo, Brazil, where the seroprevalence of toxoplasmosis is approximately 60%^14^. The study was conducted in accordance with the Declaration of Helsinki, and the Ethics Committee in Human Research at Ribeirão Preto General Hospital approved the study (approval number: 46015415.2.0000.5440). Informed consent was obtained from all adults and the legal guardians of those under 18 years of age. A detailed clinical description of patients and their ocular disease has been reported^15^. For this study, the inclusion criterion was active TRC in retinal zone 1 or 2, and the exclusion criteria were co-existing retinal pathology and media opacity preventing visualization of the posterior segment. Expanded definitions are provided in the relevant sections of the methods.

### Ophthalmic examination and diagnosis

Patients had a complete ophthalmic examination, including measurement of best-corrected Snellen visual acuity and dilated fundus examination. Toxoplasmic retinochoroiditis was classified by clinical and serological criteria. Lesions were grouped into primary (active retinochoroiditis without chorioretinal scarring), recurrent (active retinochoroiditis with chorioretinal scarring) and inactive (chorioretinal scarring alone). Only individuals with active TRC were included in this study, and any patient with co-existing, unrelated retinal pathology was excluded. Active TRC was defined as a focus of retinochoroiditis with a positive serological test for *T. gondii* immunoglobulin (Ig)G and/or IgM. Serological testing of human immunodeficiency virus (HIV) IgG was also performed. In cases of atypical toxoplasmosis that suggested the possibility of a different diagnosis, ocular fluid was tested for *T. gondii* DNA by polymerase chain reaction (PCR). Individuals who were HIV-positive had serological testing for syphilis. Documented medical history recording the timing of infection was required to classify TRC as congenital or postnatally acquired. Zone of disease was designated by location of the posterior edge of the lesion, per Holland et al.^16^. Zone 1 lesions extended to within 3000 µm of the fovea and/or 1500 µm from the optic disc margin. Zone 2 lesions were located between zone 1 and the equator. Zone 3 lesions were positioned anterior to the equator, and were excluded as SD-OCT was not possible.

### Optical coherence tomographic imaging and interpretation

The posterior pole and lesion were imaged by color photography and SD-OCT, as permitted by media clarity. Spectral domain OCT scans were acquired using the SPECTRALIS HRA + OCT (Heidelberg Engineering, Heidelberg, Germany) and analyzed with Heidelberg Eye Explorer software version 6.9.4.0. Automatic real-time tracking mode was selected, with 24 frames per location, using volume scans of the macula and lesion (20 × 15 degree area formed by 19 B-scans, each separated by 242 µm), single slice acquisition, and enhanced-depth imaging (EDI) in some cases.

An SD-OCT scan was considered gradable if the media were sufficiently clear to allow visualization of the posterior segment, as judged on resolution at the vitreoretinal interface. Patients whose presentation scans were not gradable were excluded from the study. Vitreous, posterior hyaloid, neural retina, retinal pigment epithelium and choroid were individually assessed, and findings were reported only for those scans in which the specific tissue or layer was well visualized. The SD-OCT segmentation was corrected manually. Measurements included: macular central subfield volume (1000 µm diameter), maximum lesion thickness (measured in the center of the lesion, from internal limiting membrane to base of retinal pigment epithelium, less subretinal fluid if present), and choroidal thickness under the maximum lesion thickness. Choroidal thickness was measured with the calliper tool of the SD-OCT software, and was considered thickened if it measured at least 300 µm. One eye per patient was analyzed. If both eyes had active lesions, the eye with the most posterior lesion was selected for analysis. If bilaterally active disease was symmetrical, the right eye was selected for analysis.

### Treatment and follow-up schedule

Patients with active TRC were offered treatment. Antimicrobial drug regimens included a course (6–7 weeks) of oral trimethoprim and sulfamethoxazole (160/800 mg twice daily) or oral sulfadiazine (1 g 4-times daily) and pyrimethamine (25 mg daily). Prednisone was prescribed on a case-by-case basis depending on the presence and intensity of posterior segment inflammation, and even in immunocompromised patients, at an initial dose of 20–60 mg/day with a 4-week taper. Intravitreal and intravenous anti-parasitic drugs or corticosteroids were not used. Unless the clinical situation required earlier review, patients were re-evaluated at 2 and 6–8 weeks after presentation. In selected cases, SD-OCT of the lesion and/or macula was repeated during follow-up.

### Statistical analysis

Statistical analysis was performed in GraphPad Prism (GraphPad Software, La Jolla, CA; version 7.04). Comparisons were made by features of disease, *T. gondii* and HIV serology, and visual acuity. Statistical significance was determined using Fisher’s exact test for categorical data, and Mann Whitney U test or Student t-test for continuous data. Statistically significant differences were defined by a p-value under 0.05.

### Informed consent

Informed consent was obtained from all adults and the legal guardians of those under 18 years of age.

## Results

Of a total of 262 subjects (344 eyes) with TRC, 90 individuals (90 eyes) qualified for inclusion in this study (Table 1, Supplementary Fig. S1). Mean age at presentation was 37 years (median, 33; range, 12 to 84), and 44 participants (49%) were male. A total of 8 of 90 participants (9%) had bilateral active TRC. For 90% of persons (n = 81), timing of infection was unknown. Recurrent TRC was diagnosed in 55 participants (61%), and 35 (39%) had primary ocular disease. Lesions were located in zone 1 in 30 eyes (33%) and zone 2 in 60 eyes (67%). Fourteen people (16%) were *T. gondii* IgM-positive, and all these subjects were also *T. gondii* IgG-positive. Seven participants (8%) had evidence of HIV infection; the CD4-positive T-cell count was known for 6 of these 7 individuals, with range from 45 to 1253 cells/µl (mean, 447 cell/µl), and just one having a CD4-positive T-cell count below 200 cells/µl. None of these individuals had positive syphilis serology. Most participants were treated with trimethoprim and sulfamethoxazole (n = 63, 70%), or sulfadiazine and pyrimethamine (n = 12, 13%), plus oral prednisolone (n = 77, 85%). The mean duration of follow-up was 23.9 months (range, 1–30 months).

**Table 1:** Patient and disease characteristics (N = 90 patients).

Category	Characteristic or units	N (%) or mean (standard deviation)
Sex	Female	46 (51)
	Male	44 (49)
Age	Years	37.8 ± 16.3
Race	Black	31 (34)
	Mixed race	13 (14)
	White	46 (51)
Time of infection	Congenital	1 (1)
	Postnatally acquired	8 (9)
	Unknown	81 (90)
Laterality	Bilateral disease	8 (9)
	Right eye	49 (54)
Location*	Zone 1	30 (33)
	Zone 2	60 (67)
Disease type	Primary	35 (39)
	Recurrent active	55 (61)
Ocular fluid testing	Anterior chamber paracentesis	6 (7)
	Vitreous biopsy	3 (3)
Baseline visual acuity	BCVA ≥ 20/40	33 (37)
	BCVA ≤ 20/50	26 (29)
	BCVA ≤ 20/200	31 (34)
Serology	*T. gondii* IgM-positive	14 (16)
	*T. gondii* IgG-positive	90 (100)
	HIV IgG-positive	7 (8)
Treatment	Trimethoprim + sulfamethoxazole	63 (70)
	Sulfadiazine + pyrimethamine	12 (13)
	Oral prednisolone	77 (85)
	No treatment	6 (7)

BCVA = best corrected visual acuity; Ig = immunoglobulin.

*Location: Zone 1 = 3000 µm from fovea plus 1500 µm from disc margin; Zone 2 = between equator and zone 1.

**Figure 1:**
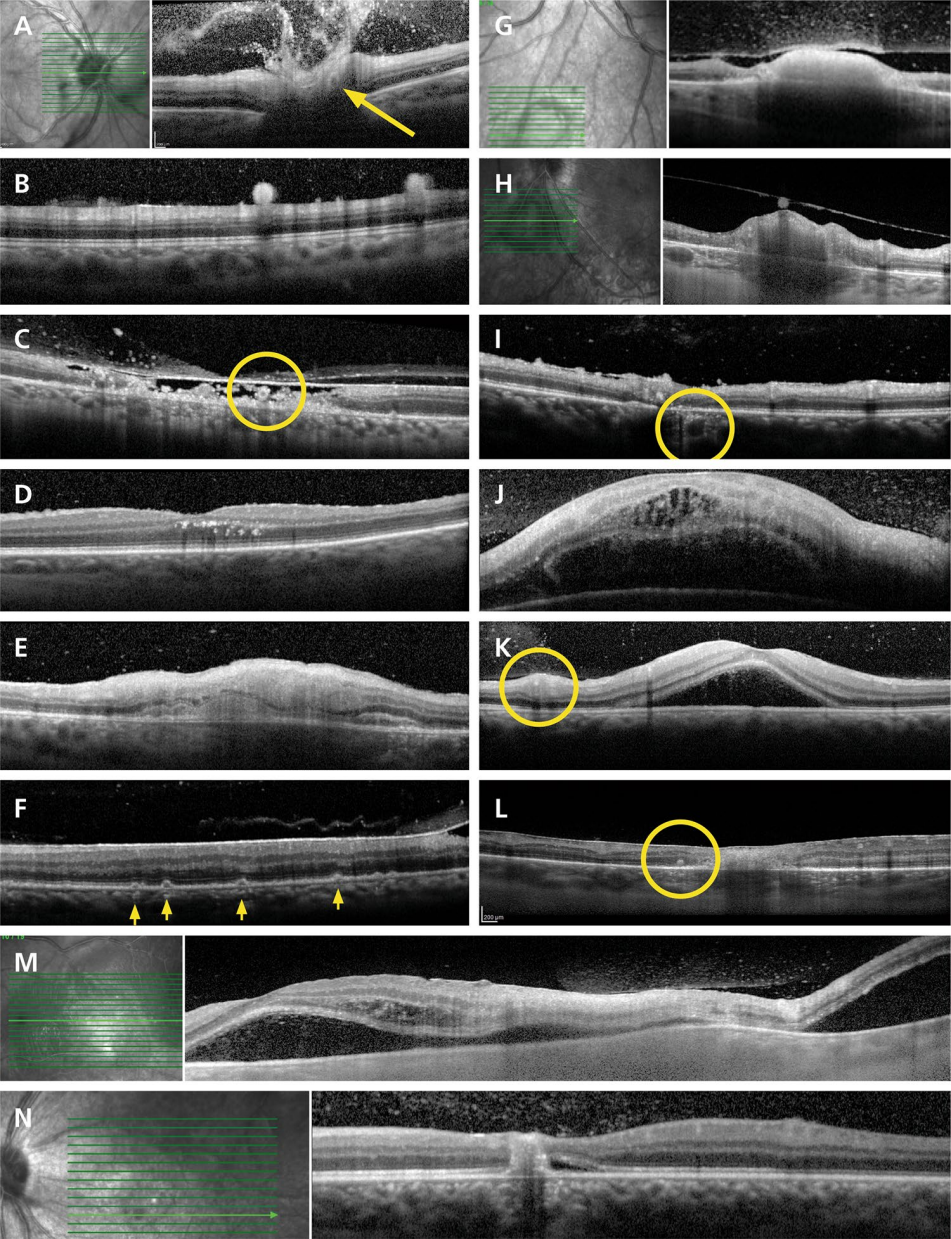
Representative signs of active toxoplasmic retinochoroiditis by spectral domain optical coherence tomography. (**A**) Hyperreflective dots in the vitreous cavity overlying the optic disc (zone 1, primary, IgM-negative); note perivascular hyperreflectivity (arrow); (**B**) Large hyperreflective deposits (190 µm) at vitreoretinal interface (zone 2, recurrent, IgM-negative); (**C**) Hyperreflective blood vessel (circled, identified on en-face image) within a hyporeflective retinal space (HIV-positive, zone 2, recurrent, IgM-negative); (**D**) Intraretinal hyperreflective dots in the outer plexiform and outer nuclear layers (zone 2, primary, IgM-positive); (**E**) “Ribbon” appearance of hyporeflective outer nuclear layer between thickened hyperreflective photoreceptor-RPE complex and hyperreflective inner nuclear layer (zone 1, recurrent, IgM-negative); (**F**) RPE “bumps” (arrows), (HIV-positive, zone 2, recurrent, IgM-negative); (**G**) Hyperreflective dots in the vitreous cavity, thickened posterior hyaloid with partial posterior vitreous detachment; retinal thickening with full-thickness hyperreflectivity and adjacent disorganization of the retinal layers; adjacent RPE thickening, choroidal hyporeflectivity and thickening (zone 2, primary, IgM-negative); (**H**) Large hyperreflective deposit on posterior hyaloid face (zone 2, recurrent, IgM-negative); note thickened hyperreflective retina with choroidal hyporeflectivity and thickening; (**I**) Hyperreflective dots surrounding choroidal vessel (circled); hyperreflective dots on internal limiting membrane and disorganized retinal layers (zone 2, recurrent, IgM-negative); (**J**) Gross macular thickening with hyperreflective dots and intraretinal fluid in the outer plexiform and outer nuclear layers, subretinal fluid and bowing of RPE-Bruch’s membrane complex (zone 1, primary, IgM-negative); (**K**) Subfoveal fluid and hyperreflective dots in the vitreous cavity overlying a blood vessel (circled) (zone 2, primary, IgM-negative); (**L**) Round body in outer nuclear layer (circled), with adjacent retinal hyperreflectivity and disorganization of retinal layers (zone 2, primary, IgM-positive); (**M**) Subfoveal fluid with hyperreflective retinal dots, outer plexiform and outer nuclear layer fluid and hyperreflective dots, retinal hyperreflectivity, bowing of RPE-Bruch’s membrane complex, overlying hyperreflective dots in the vitreous cavity and partial separation of posterior hyaloid (zone 2, primary, IgM-negative); (**N**) Hyperreflective dots in the vitreous cavity, disorganization of retinal layers, RPE thickening with subjacent choroidal hyporeflectivity and adjacent subfoveal fluid (zone 1, recurrent, IgM-negative). Abbreviations: SD-OCT = spectral domain optical coherence tomography, TRC = toxoplasmic retinochoroiditis, Ig = immunoglobulin, RPE = retinal pigment epithelial/epithelium, HIV = human immunodeficiency virus.

At presentation, the majority of SD-OCT signs involved the retina (Supplementary Fig. S2), which was thickened at the site of TRC relative to adjacent uninvolved retina in two-thirds of cases (n = 46/71, 65%) (Table 2, Fig. 1). This was more common in zone 1 lesions, which were on average 201 µm thicker than zone 2 lesions (*p* < 0.01). Retinal hyperreflectivity (with the hyporeflective inner and outer nuclear layers becoming hyperreflective, resulting in full-thickness hyperreflectivity) was a presenting feature in 43 of 70 lesions (61%), and more likely in zone 1 lesions or primary disease (*p* < 0.05). Disorganization of the retinal layers was evident in 16 of 71 lesions (23%), and noted more often in zone 2 or recurrent disease (*p* < 0.05). Intraretinal hyperreflective dots were seen in 7 of 70 lesions (10%). Large outer retinal cystic spaces were present in 7 of 73 lesions (10%).

**Table 2:** Posterior segment features on SD-OCT in active TRC at presentation for 90 eyes of 90 patients. Comparisons were made between TRC lesions located in zones 1 and 2, and primary vs. recurrent TRC. Denominator indicates number of eyes for each item. The *p* values were calculated using Fisher’s exact test or Mann Whitney U test (* *p* < 0.05, ** *p* < 0.01).

SD-OCT feature at presentation	Total/gradable scans		Location				Episode			
			Zone 1		Zone 2		Primary		Recurrent	
Mean lesion height (µm) ± standard deviation	434 ± 202		**565 ± 179**		364 ± 173 **		491 ± 219		382 ± 165	

Lesion	N	%	N	%	N	%	N	%	N	%
Involving optic disc	9/73	12	9/29	31	N/A		**8/31**	**26**	**1/42**	2 **
Thickened retina	46/71	65	**24/28**	**86**	**22/43**	51 **	23/31	74	23/40	58
Full-thickness retinal hyperreflectivity	43/70	61	**22/29**	**76**	**21/41**	51 *	**23/30**	**77**	**20/40**	50 *
Intraretinal hyperreflective dots	7/70	10	3/29	10	4/41	10	3/30	10	4/40	10
Periarteriolar hyperreflective dots	4/73	5	1/29	3	3/44	7	2/31	6	2/42	5
Round outer plexiform bodies	2/73	3	0/29	0	2/44	5	2/31	6	0/42	0
Outer nuclear ribbon	1/73	1	1/29	3	0/44	0	0/31	0	1/42	2
Disorganized retinal layers	16/71	23	**3/29**	**10**	**13/42**	31 *	**3/30**	**10**	**13/41**	32 *
Retinal hyporeflective space	4/71	6	2/29	7	2/42	5	1/30	3	3/41	7
Intraretinal fluid	1/68	1	0/28	0	1/40	3	0/31	0	1/37	3
Large outer retinal cystic spaces	7/73	10	3/29	10	4/44	9	3/31	10	4/42	10
Subretinal fluid	1/68	1	0/28	0	1/40	3	1/31	3	0/37	0
RPE thickening / bumps	14/26	54	6/8	75	8/18	44	4/8	50	10/18	56
RPE atrophy	9/26	35	2/8	25	7/18	39	3/8	38	6/18	33
Bowing of retina-RPE-Bruch’s membrane	6/73	8	1/29	3	5/44	11	1/31	3	5/42	12
Choroidal hyporeflectivity / shadowing	25/41	61	8/11	73	17/30	57	9/14	64	16/27	59
Choroidal hyperreflectivity	7/41	17	0/11	0	7/30	23	2/14	14	5/27	19
Choroidal thickening	22/40	55	6/11	55	16/29	55	5/12	42	17/28	61

Adjacent to lesion	N	%	N	%	N	%	N	%	N	%
Disorganized retinal layers	8/73	11	2/29	7	6/44	14	5/31	16	3/42	7
Subretinal or intraretinal fluid	11/73	15	6/29	21	5/44	11	**8/31**	**26**	**3/42**	7 *
Hyperreflective intraretinal dots	9/73	12	5/29	17	4/44	9	**7/31**	**23**	**2/42**	5 *
Choroidal perivascular hyperreflective dots	4/73	5	1/29	3	3/44	7	2/31	6	2/42	5

Vitreous	N	%	N	%	N	%	N	%	N	%
Normal appearance	16/80	20	7/29	24	9/51	18	7/32	22	9/48	19
Hyperreflective dots	64/80	80	22/29	76	42/51	82	25/32	78	39/48	81
Vitreoschisis	2/80	3	1/29	3	1/51	2	2/32	6	0/48	0
Hyperreflective deposits ≥ 25 µm	29/80	36	8/29	28	21/51	41	14/32	44	15/48	31
Posterior hyaloid thickening over lesion	28/80	35	9/29	31	19/51	37	10/32	31	18/48	38
Posterior hyaloid attached at lesion	70/80	88	26/29	90	44/51	86	**31/32**	**97**	**39/48**	81*
Traction adjacent to lesion	1/80	1	0/29	0	1/51	2	0/32	0	1/48	2
Partial posterior vitreous detachment	13/80	16	**1/29**	**3**	**12/51**	24*	4/32	13	9/48	19
Epiretinal membrane at lesion	4/80	5	0/29	0	4/51	8	1/32	3	3/48	6

SD-OCT = spectral domain optical coherence tomography; TRC toxoplasmic retinochoroiditis; RPE = retinal pigment epithelial/epithelium.

Retinal changes adjacent to the lesion included disorganization of the retinal layers in 8 of 73 eyes (11%), subretinal or intraretinal fluid in 11 of 73 eyes (15%), and hyperreflective intraretinal dots in 9 of 73 eyes (12%). Optic disc lesions (n = 9/73, 12%) were more common in primary disease (*p* < 0.01), which also was associated with adjacent subretinal or intraretinal fluid (*p* < 0.05). Retinal hyporeflective spaces (signal voids within the retina, extending between the internal limiting membrane and retinal pigment epithelium) were noted in 4 of 71 lesions (6%) and periarteriolar hyperreflective dots were present in 4 of 73 lesions (5%).

Novel retinal findings included round outer plexiform bodies (n = 2/73, 3%) and a “ribbon-like” outer nuclear layer, created when inner nuclear layer became hyperreflective while the outer nuclear layer retained its hyporeflectivity (n = 1/73, 1%) (Table 2, Fig. 1). The retinal pigment epithelium at the lesion was visible in 26 SD-OCT scans at presentation, and showed thickening in half. Atrophic epithelial changes were evident in 9 of 26 eyes (35%). Retinal pigment epithelial “bumps” were present in one patient with concurrent HIV infection (Fig. 1F). Forty of 90 patients had an SD-OCT scan with EDI at presentation, and the choroid was thickened in 55% of these scans (n = 22). Choroidal hyporeflectivity was a common presenting feature, present in 61% of eyes (n = 25/41 eyes), and choroidal perivascular hyperreflective dots were identified in 5% of eyes (n = 4/73). Round bodies in the outer plexiform layer was the only retinal or choroidal sign associated with *T. gondii* IgM-positive TRC (*p* < 0.05) and there were no differences between retinal or choroidal signs in HIV-positive versus -negative patients (Supplementary Table S1).

**Figure 2:**
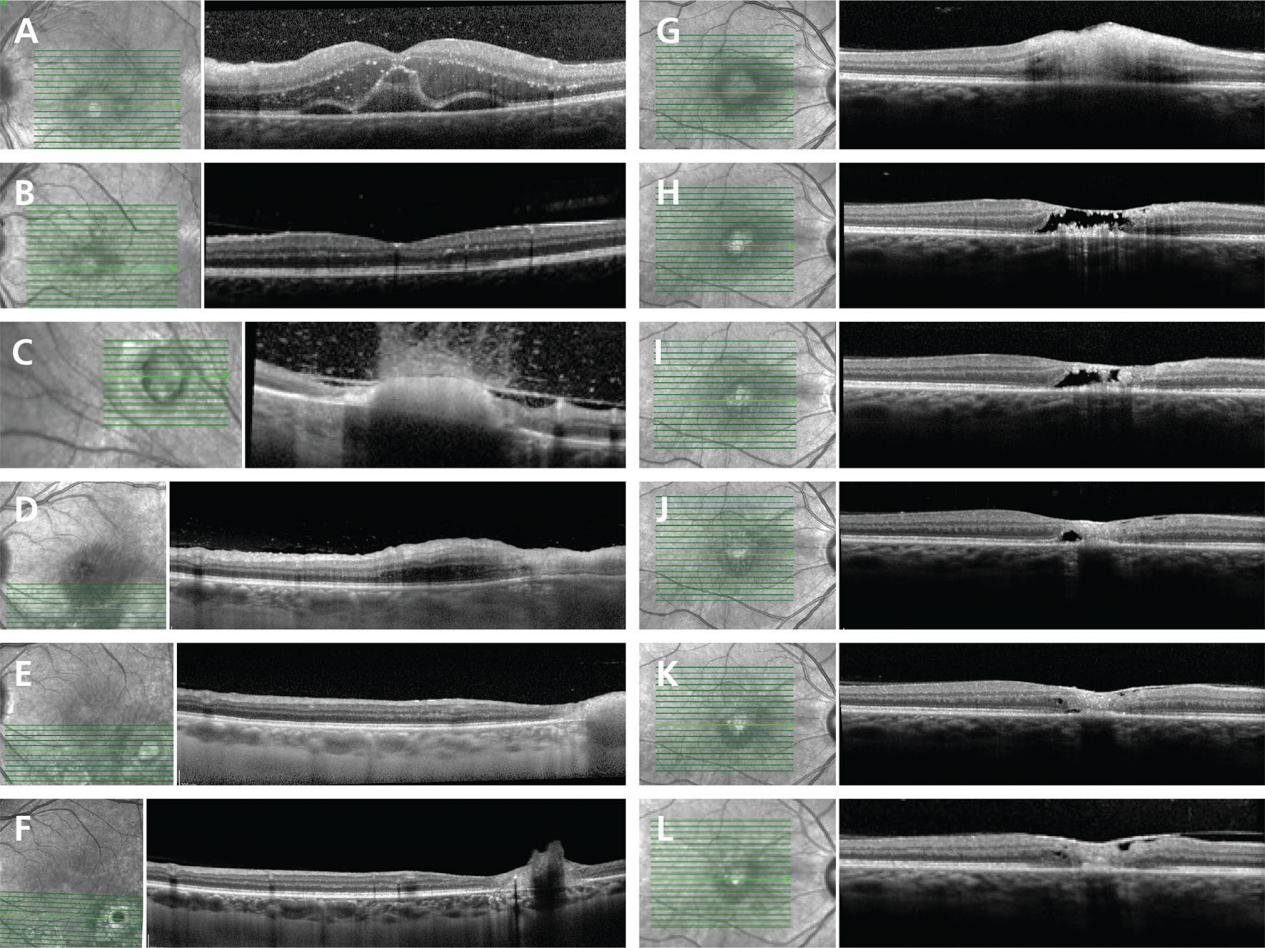
Macular oedema and evolution of retinal necrosis in TRC. (**A**-**C**) Macular oedema in TRC. (**A**) Patient presents with a superonasal focus of TRC in the left fundus, with hyperreflective dots in the vitreous cavity, small hyperreflective dots in outer plexiform layer, outer nuclear and photoreceptor layers, and marked intraretinal fluid at the macula with subfoveal fluid. Small hyperreflective dots are especially prominent in the outer plexiform and outer nuclear layers; (**B**) At 8 weeks post-presentation, the macular oedema had resolved, with widespread small hyperreflective intraretinal dots; (**C**) Lesion in zone 2 at 8 weeks after presentation. There are hyperreflective dots in the vitreous cavity, posterior hyaloid thickening and separation, full thickness retinal hyperreflectivity and subjacent choroidal hyporeflectivity. (**D**–**F**) Evolution of “coagulative necrosis" in TRC. SD-OCT scans in a patient with recurrent, IgM-negative TRC. (**D**) At presentation, there is full-thickness retinal hyperreflectivity, adjacent retinal thickening, preservation of retinal layers and outer retinal oedema; (**E**) At 4 weeks after presentation, revealing retinal hyperreflectivity with pronounced choroidal hyporeflectivity. Note adjacent retinal thinning, disorganization, and RPE hyporeflectivity with choroidal shadowing; (**F**) At 6 months after presentation, dense retinal hyperreflectivity associated with choroidal shadowing and RPE atrophy. (**G**-**L**) Evolution of “liquefactive necrosis” in TRC**.** SD-OCT scan of right macula in a patient presenting with zone 1, primary *T. gondii* IgM-positive TRC. (**G**) At presentation, demonstrating full-thickness retinal hyperreflectivity, thickening and loss of retinal organization. Note lack of hyperreflective dots in the vitreous cavity; (**H**) At 7 days post-presentation. Necrosis demonstrated by large retinal hyporeflective space within retina and preservation of the ILM, and RPE thickening; (**I**) At 2 weeks after presentation, hyporeflective space is smaller; (**J**) Week 5 after presentation, disorganized retinal tissue and RPE thickening; (**K**) Nine weeks after presentation, necrosis has resolved and is replaced with granular disorganized tissue; (**L**) At 4 months post-presentation, showing partial separation of the posterior hyaloid, ERM formation, disorganized retinal tissue, thickened RPE and focal choroidal hyporeflectivity. Abbreviations: SD-OCT = spectral domain optical coherence tomography, TRC = toxoplasmic retinochoroiditis, Ig = immunoglobulin, RPE = retinal pigment epithelial/epithelium, ILM = internal limiting membrane, ERM = epiretinal membrane.

Changes at the macula on SD-OCT were evident in 64 of 86 eyes (74%) at presentation (Table 3). Zone 1 lesions were associated with hyperreflective dots at the vitreoretinal interface (n = 6/29, 21%), full-thickness retinal hyperreflectivity (n = 5/29, 17%), and submacular choroidal thickening (n = 6/29, 21%) (*p* < 0.01). Intraretinal hyperreflective dots (n = 8/29, 28%) and hyporeflective retinal spaces (n = 3/29, 10%) were associated with zone 1 TRC (*p* < 0.05). Active TRC in zone 2 was associated with an abnormal macular scan in 22 of 57 eyes (39%). Macular oedema was seen in 19 of 86 eyes (22%), irrespective of zone of lesion (Fig. 2A-C). Epiretinal membrane was observed in 10 of 86 eyes (12%), and vitreomacular traction was an uncommon presenting feature (n = 3/86, 3%).

**Table 3:** Macular findings on SD-OCT in active TRC at presentation for 86 eyes (86 patients). Comparison was made between TRC lesions located in zones 1 and 2. The *p* values were calculated using Fisher’s exact test or Student t-test (* *p* < 0.05, ** *p* < 0.01, *** *p* < 0.001).

SD-OCT feature at macula	Total N (%)	Zone 1 N (%)	Zone 2 N (%)
Total	86	29	57
Normal appearance	22 (26)	**0 (0)**	22 (39) ***
Hyperreflective dots at vitreoretinal interface	6 (7)	**6 (21)**	0 (0) **
Vitreomacular traction	3 (3)	0 (0)	3 (5)
Epiretinal membrane	10 (12)	1 (3)	9 (16)
Full-thickness retinal hyperreflectivity	5 (6)	**5 (17)**	0 (0) **
Hyperreflective dots retina	13 (15)	**8 (28)**	5 (9) *
Macular oedema	19 (22)	8 (28)	11 (19)
Hyporeflective retinal space	3 (3)	**3 (10)**	0 (0) *
Subretinal fibrosis	1 (1)	1 (3)	0 (0)
RPE thickening	2 (2)	0 (0)	2 (4)
RPE detachment	1 (1)	0 (0)	1 (2)
Choroidal thickening	7 (8)	**6 (21)**	1 (2) **
Central subfield volume (mm^3^) ± standard deviation	0.28 ± 0.11 (N = 76)	0.29 ± 0.14 (N = 23)	0.27 ± 0.10 (N = 53)

SD-OCT = spectral domain optical coherence tomography; TRC = toxoplasmic retinochoroiditis; RPE = retinal pigment epithelial/epithelium.

Presenting SD-OCT findings in the vitreous included hyperreflective dots (n = 64/80 eyes, 80%), although these were not associated with lesion location, disease type, *T. gondii* IgM positivity and serologic evidence of HIV infection (Table 2, Supplementary Table S1). The posterior hyaloid was attached at the lesion in 70 of 80 eyes (88%), and this was more common in primary TRC (*p* < 0.05). A partial posterior vitreous detachment was noted in 13 of 80 eyes (16%), and occurred more often in zone 2 TRC (*p* < 0.05). Posterior hyaloid thickening and large hyperreflective deposits at the vitreoretinal interface were present in approximately one-third of eyes (n = 28/80 and 29/80, respectively) at presentation, and there was an epiretinal membrane across 4 of 80 lesions (5%).

Eyes of selected patients, followed for at least 8 weeks without recurrence, had additional SD-OCT imaging of the lesion and/or macula during follow-up (Supplementary Table S2). Common signs included disorganization of retinal layers (n = 11/15, 73%) and hyaloid thickening (n = 8/15, 53%). Involved retina often became thinned relative to adjacent tissue (n = 6/15, 40%), while the retinal pigment epithelium became thickened at (n = 6/15 eyes, 40%) or adjacent to (n = 3/15 eyes, 20%) the lesion. Macular epiretinal membrane developed in 10 of 23 eyes (43%).

Two types of retinal destruction were visualized by SD-OCT. The most common form of necrosis presented with full-thickness retinal hyperreflectivity and thickening, and a well-demarcated border to adjacent normal-appearing retina, with underlying choroidal hyporeflectivity (presumed to represent shadowing in the majority of cases) and hyperreflective dots in the vitreous (Fig. 2D-F, interpreted as coagulative retinal necrosis). Over time, the lesion progressively thinned, and became disorganized and less hyperreflective. Less commonly, retinal destruction presented as hyporeflective spaces or signal voids within the neural retina, and preservation of the retinal pigment epithelium and internal limiting membrane (Fig. 2G-L, interpreted as liquefactive retinal necrosis). There were relatively fewer hyperreflective dots in the vitreous, and hyporeflective retinal spaces were slowly replaced by disorganized retinal tissue in follow-up.

Features of active TRC by SD-OCT were compared to visual outcomes in eyes followed for at least 8 weeks (n = 47) (Table 4). The only SD-OCT finding associated with visual outcome was retinal destruction with hyporeflective spaces or signal voids, which was unusual in eyes achieving visual acuities of 20/40 or better, and present in half of eyes retaining visual acuities of 20/200 or worse (n = 1/25 and 7/14, respectively, *p* < 0.01). No associations were found between visual outcomes and the presence of large hyperreflective dots (n = 15/47, 32%), intraretinal or macular oedema (n = 9/47, 29%), posterior hyaloid thickening (n = 21/47, 45%) and macular epiretinal membrane (n = 15/47, 32%).

**Table 4:** Features on SD-OCT at any time during clinical course and visual outcomes in active TRC for 47 eyes of 47 patients, who were followed for at least 8 weeks and did not have a recurrence. Comparisons were made between eyes with good visual acuity (20/40 or better) and eyes with poor visual acuity (20/200 or worse). The p-values were calculated using Fisher’s exact test (** *p* < 0.01).

SD-OCT feature during active TRC	Best corrected visual acuity at resolution					
	Total		> 20/40		< 20/200	
	N	%	N	%	N	%
Hyperreflective dots ≥ 25 µm	15/47	32	6/25	24	6/14	43
Hyporeflective retinal space	8/47	17	**1**/25	**4**	**7**/14	50 **
Macular oedema	9/47	29	3/25	12	3/14	21
Posterior hyaloid thickening	21/47	45	13/25	52	7/14	50
Macular ERM	15/47	32	6/25	24	6/14	43

SD-OCT = spectral domain optical coherence tomography; TRC = toxoplasmic retinochoroiditis; ERM = epiretinal membrane.

## Discussion

In this large cohort of patients presenting with TRC, SD-OCT of the lesion frequently demonstrated thickened hyperreflective retina, choroidal thickening and hyporeflectivity, hyperreflective dots in the vitreous and posterior hyaloid thickening. Adjacent disorganization of the retinal layers was often seen. Over time, the retina thinned and became less hyperreflective, but did not return to a normal appearance, and there was retinal pigment epithelial thickening with choroidal hyporeflectivity, or epithelial atrophy with choroidal hyperreflectivity.

The necrotizing retinitis that defines active TRC clinically represents tissue destruction related to proliferation of *T. gondii* and the resultant inflammation^17^. Coagulative necrosis is the most common form of the process, characterized by preservation of tissue architecture. In TRC, this is likely represented as full-thickness retinal hyperreflectivity on SD-OCT. For 5% of eyes, the presentation SD-OCT showed hyporeflective retinal spaces or signal voids with an intact internal limiting membrane. A similar finding was described in the case report of TRC imaged by SD-OCT, and was assumed to represent liquefactive necrosis^18^. While there are obvious difficulties in providing a retinal biopsy to correlate SD-OCT and clinical findings in TRC, the technique of OCT-mounted core biopsy in oncology has been used to confirm that liquefactive necrosis appears as hyporeflective tissue spaces^19^. Liquefactive necrosis probably occurs where there is heavy parasite proliferation with massive cell lysis, and profound tissue destruction. We observed hyporeflective retinal spaces in 17% of eyes over the course of TRC, and found this to be predictive of poor visual outcome.

Subretinal or intraretinal fluid, usually adjacent to the lesion, was observed in approximately 15% of eyes presenting with active TRC, and was more common in primary disease. Published SD-OCT studies have identified subretinal fluid in as many as 43% of patients^20^. Two studies from the same group documented intraretinal and subretinal fluid in SD-OCT scans of TRC, although the authors did not explore clinical associations^10,11^. An unusual presentation of outer retinal oedema—“huge outer retinal cystic spaces”^11^—was identified in two of eyes in our series. This form of retinal oedema usually occurs at the macula, affects the outer plexiform, outer nuclear and photoreceptor layers, and is accompanied by subretinal fluid. Macular oedema was a presenting SD-OCT sign in approximately 20% of eyes; this was equally frequent in zone 1 and zone 2 lesions, highlighting that zone 2 TRC is not a benign process. Macular epiretinal membrane was noted during follow-up in 40% of eyes in our series.

Vitritis is a common clinical feature of TRC, represented as hyperreflective dots in the vitreous cavity on SD-OCT. Large hyperreflective deposits (over 25 µm) were seen in the vitreous of one-third of eyes at presentation. This feature has been noted in both viral and parasitic retinal necrosis, and absorption of these deposits into the retina has been documented with both time domain OCT and SD-OCT^8,13,21,22^. The deposits may represent clumps of inflammatory cells and/or parasite cysts^23^. In one report of an individual with severe TRC, the authors found clusters of macrophages attached to the posterior hyaloid and internal limiting membrane in the enucleated globe^24^. Encysted *T. gondii* has been isolated from the vitreous of patients with TRC who required diagnostic vitrectomy^23,25–27^. Cysts contain multiple dormant parasites, and measure 20 to 40 µm in diameter^26^.

Small round outer nuclear hyperreflective bodies (approximately 80 µm) was an unusual SD-OCT sign that we observed in two *T. gondii* IgM-positive patients. These bodies might represent encysted parasites, although histopathological reports indicate cysts are most commonly found in the ganglion cell layer. We otherwise found no distinction between SD-OCT features of *T. gondii* IgM-positive versus -negative TRC. Although differences between IgM-positive and -negative disease have been speculated, a study of post-mortem-enucleated globes with TRC showed IgM serology did not distinguish pathological features, including extension of necrosis and number of cysts^28^. Aggressive TRC has been associated with acquired immunodeficiency syndrome^29^. Our study suggested SD-OCT signs were not impacted by HIV infection status, although retinal pigment epithelial “bumps” and retinal arteriolar hyperreflective were identified in one eye of an HIV-positive patient and no other cases. These RPE bumps were observed in 12 of 13 cases of active TRC cases in a recent retrospective study^30^. Since only one HIV-positive patient had a CD4-positive T cell count below 200 cells/µl, it was not possible to evaluate associations between OCT signs and counts.

One previous study of SD-OCT in TRC specified 71% of gradable scans, which is comparable to our rate of 68%^7^. However, in contrast to this and other studies, we observed a relatively low prevalence of signs. As one example, rates of hyaloid thickening were 90–95% in other studies, compared to just 35% in our study^7,12^. As other examples, we recorded lower rates of large hyperreflective deposits and focal hyaloid attachment than were identified in these studies (i.e. 50–60% vs. 36%, and 50% vs. 16%, respectively)^7,13^. Our patient group was considerably larger than any other studied to date. In addition, we likely had little referral bias in this work; over 75% of the population uses public healthcare in Brazil, and thus the Uveitis Service at Ribeirão Preto General Hospital serves a comprehensive patient population^31^.

Recently, SD-OCT-angiography has been used for evaluation of active TRC, showing hyporeflective signals at the level of superficial and deep capillary retinal plexus, as well as in choriocapillaris and Sattler layers, corresponding to nonperfused or hypoperfused areas^20^. Partial blood flow recovery occurs in most cases following resolution of an active lesion^32^. Intraretinal vascular abnormalities^32,33^, retinal^32^ and choroidal neovascularization^20^ also are reported SD-OCT-angiographic features of TRC.

We present the largest prospective cohort of individuals with active TRC studied by SD-OCT. Given the challenge of diagnosing primary TRC in particular, one of our goals was to identify diagnostic features. A broad spectrum of SD-OCT signs was identified in patients with active TRC, but no sign was universal. All the posterior segment SD-OCT signs we observed have been reported in other forms of infectious and non-infectious uveitis^13,34,35^. Another goal was to identify prognostic signs: we have showed that hyporeflective retinal spaces —probably representing liquefactive necrosis —is associated with poor visual outcome. Future refinements in the capacity of OCT to resolve tissue architecture may identify additional signs to aid in the management of TRC.

## Supplementary Information

Supplementary Information.
