# Supplementary material for: Posterior segment findings by spectral-domain optical coherence tomography and clinical associations in active toxoplasmic retinochoroiditis
Source: Sci Rep. 2022 Jan 21;12:1156. doi: 10.1038/s41598-022-05070-9 (PMC8782858; doi:10.1038/s41598-022-05070-9)
Supplement: Supplementary file 1 — Supplementary Information. [file 41598_2022_5070_MOESM1_ESM.pdf]

Supplementary Figure S1. Schematic representation of study selection criteria. Data were available from 262 patients presenting consecutively at a tertiary-referral uveitis clinic with *T. gondii* IgG-positive TRC who were studied with Heidelberg SPECTRALIS SD-OCT of the posterior segment of the eye. Exclusions are indicated, resulting in a final cohort of 90 eyes with active TRC.

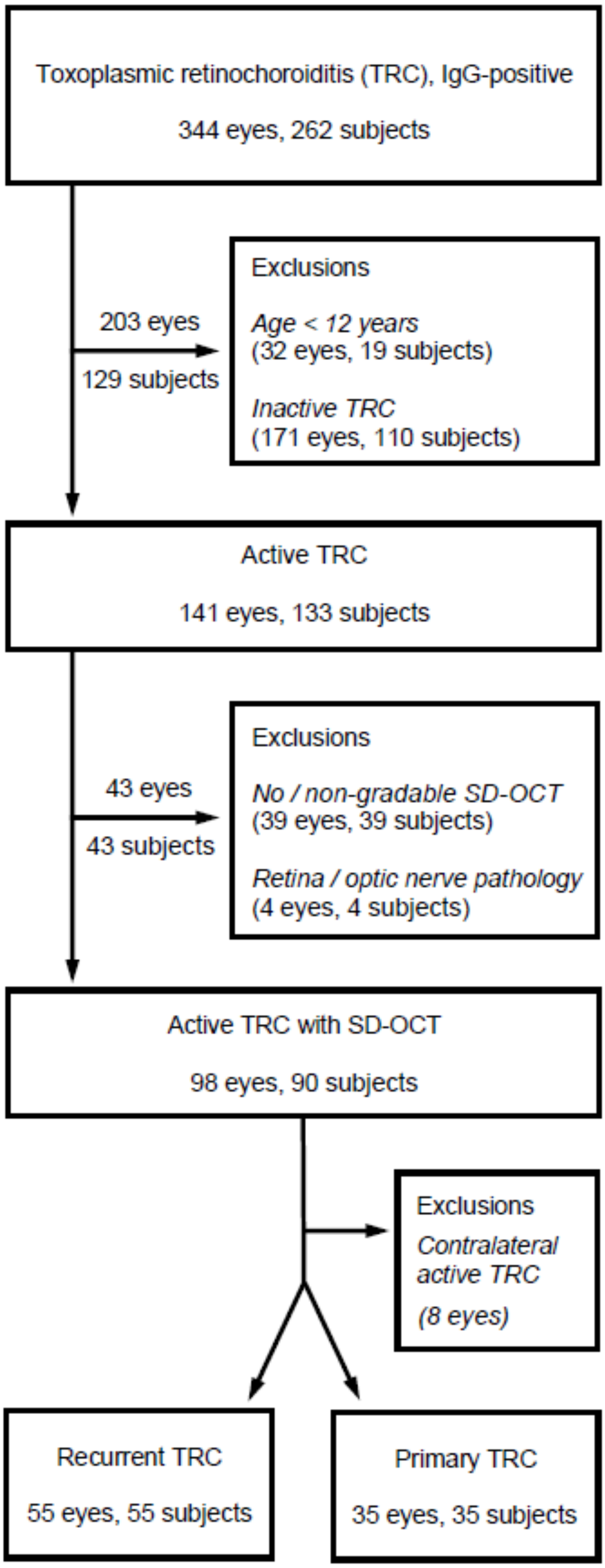

Supplementary Figure S2. Fundus photography and SD-OCT in active toxoplasmic retinochoroiditis. Representative images from patients presenting to a tertiary referral uveitis clinic with T. gondii IgG-positive TRC who were studied with Heidelberg SPECTRALIS SD-OCT of the posterior segment of the eye. Color fundus photographs for clinical correlation. SD-OCT demonstrating A. Hyperreflective dots in the vitreous and vitreoretinal interface, thickened posterior hyaloid, full-thickness retinal hyperreflectivity, and choroidal shadowing (hyporeflectivity) and thickening in recently diagnosed active TRC. B. RPE elevation with adjacent loss of retinal layers in a healing TRC lesion. C. Epiretinal membrane, central full-thickness retinal hyperreflectivity with loss of adjacent retinal layers in a subject with active TRC undergoing antibiotic therapy. Abbreviations: Ig = immunoglobulin, TRC = toxoplasmic retinochoroiditis, SD-OCT = spectral domain optical coherence tomography, RPE = retinal pigment epithelial.

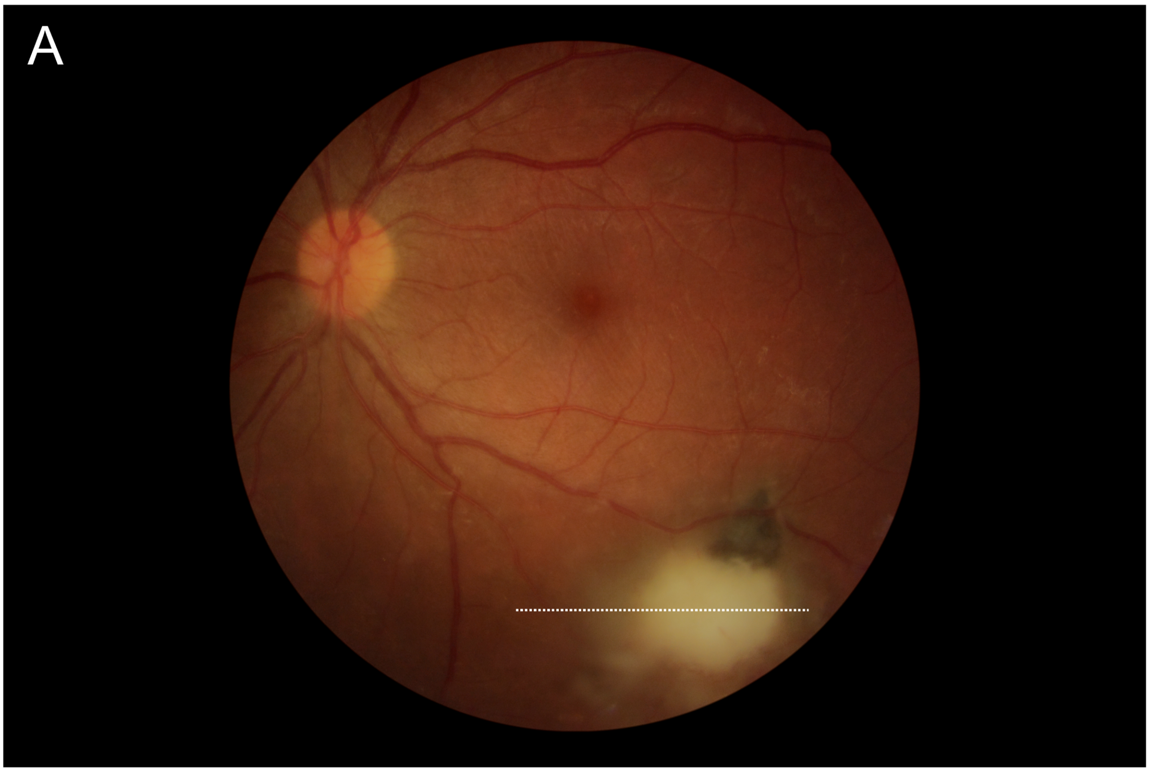

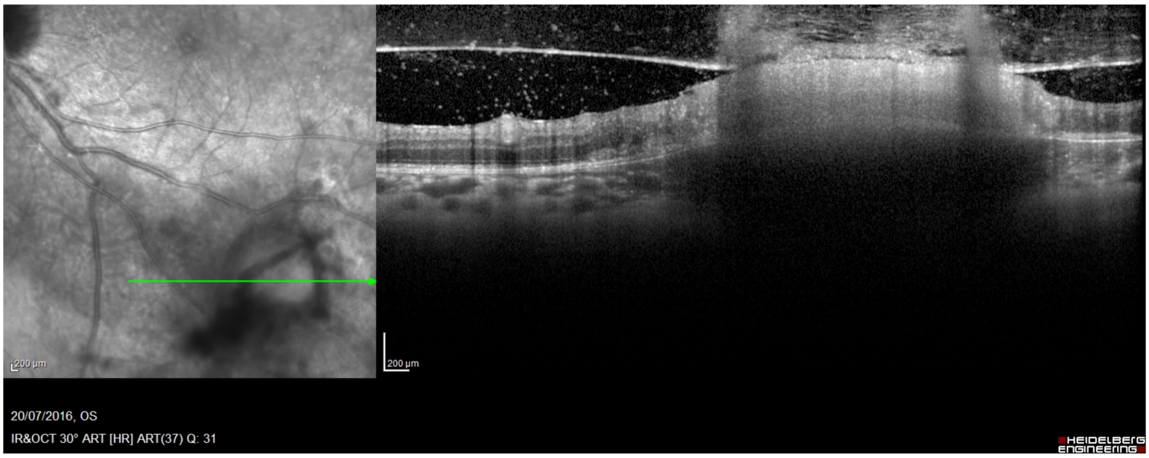

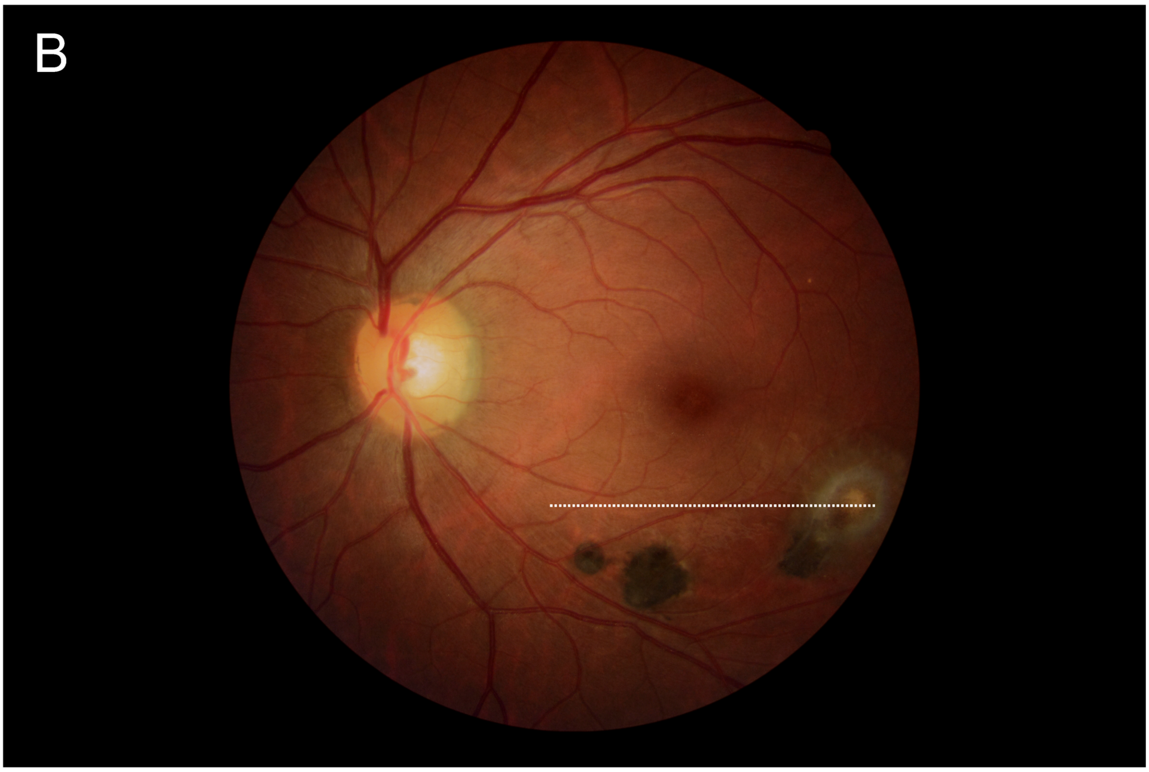
B

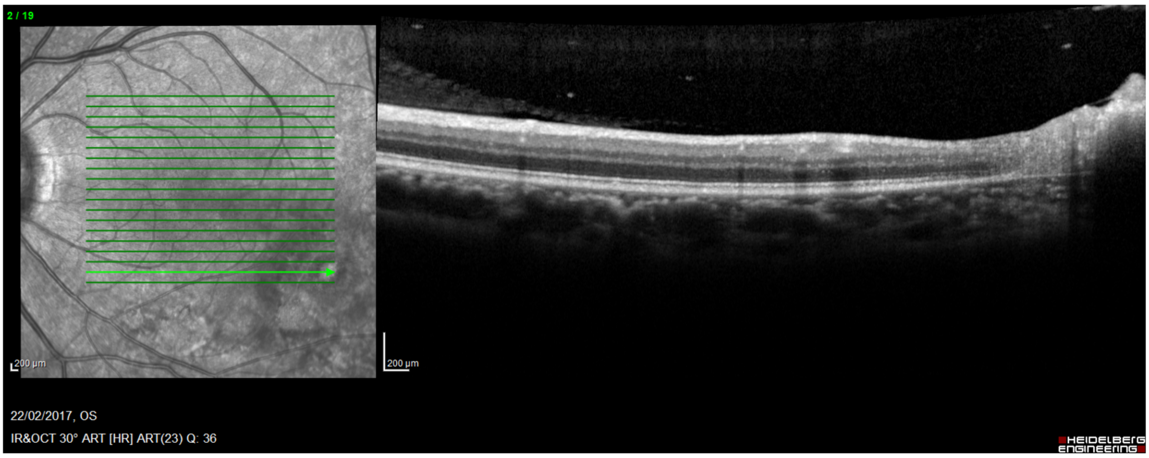
2 / 19
200 µm
200 µm
22/02/2017, OS
IR&OCT 30° ART [HR] ART(23) Q: 36
HEIDELBERG ENGINEERING

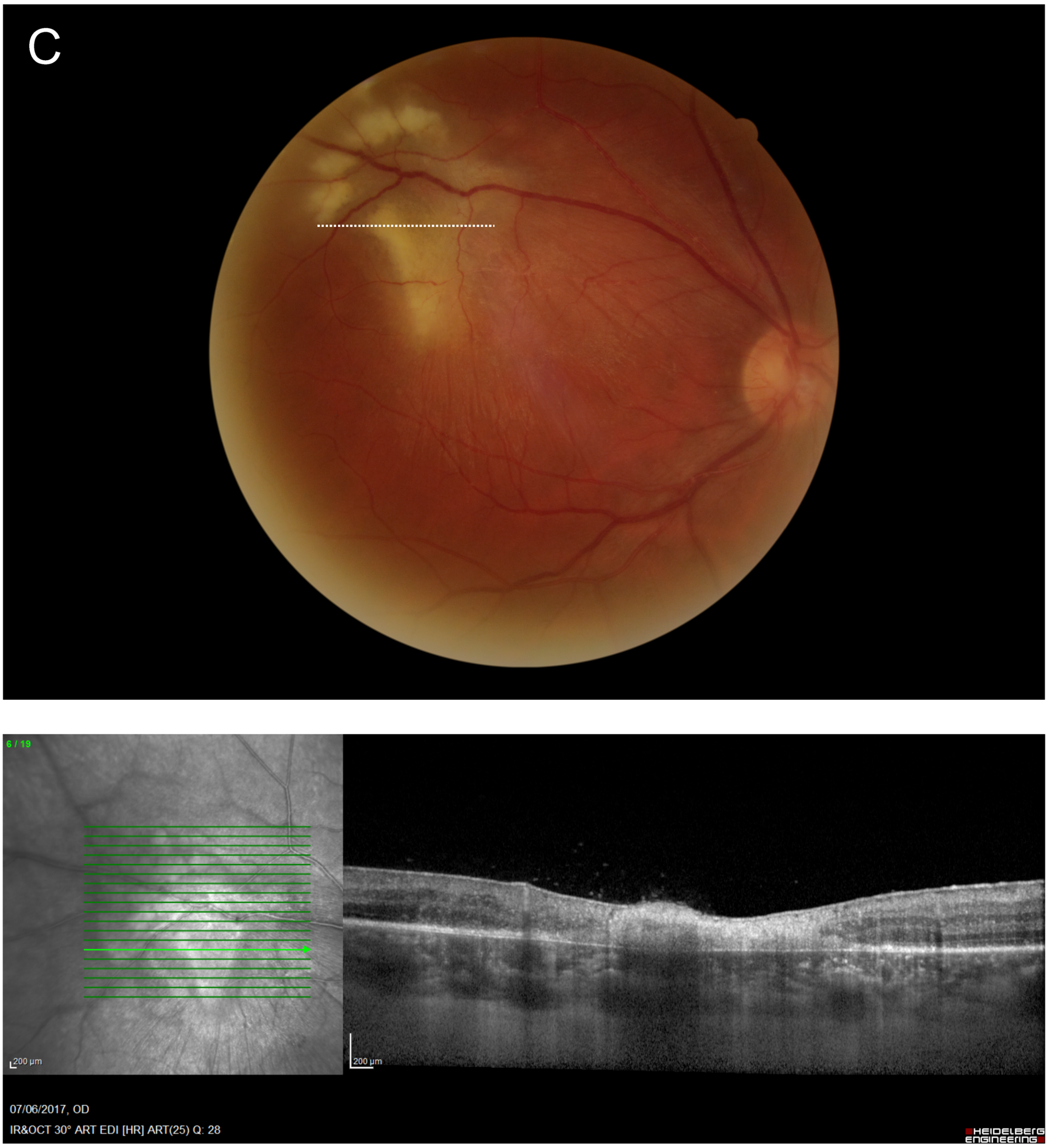
C
6 / 19
200 µm
200 µm
07/06/2017, OD
IR&OCT 30° ART EDI [HR] ART(25) Q: 28

Supplementary Table S1. Posterior segment features on SD-OCT in active TRC at presentation for 90 eyes of 90 patients. Comparisons were made between *T. gondii* IgM status and HIV infection. Denominator indicates number of eyes for each item. The p-values were calculated using Fisher's exact test or Mann Whitney U test (* p < 0.05).

SD-OCT feature at presentation	Total / gradable scans		*T. gondii* serology				HIV infection			
			IgM +		IgM –		HIV +		HIV –	
Mean lesion height (μm) ± standard deviation	434 ± 202		463 ± 179		424 ± 205		406 ± 213		435 ± 199	
LESION	N	%	N	%	N	%	N	%	N	%
Involving optic disc	9/73	12	0/14	0	9/59	15	1/7	14	8/66	12
Thickened retina	46/71	65	10/14	71	36/57	63	3/7	43	43/64	67
Full-thickness retinal hyperreflectivity	43/70	61	9/14	65	34/56	61	3/6	50	40/64	63
Intraretinal hyperreflective dots	7/70	10	1/14	7	6/56	11	0/6	0	7/64	11
Periarteriolar hyperreflective dots	4/73	5	0/14	0	4/59	7	1/7	14	3/66	5
Round outer plexiform bodies	2/73	3	**2/14**	**14**	**0/59**	**0 ***	1/7	14	1/66	2
Outer nuclear ribbon	1/73	1	0/14	0	1/59	2	0/7	0	1/66	2
Disorganized retinal layers	16/71	23	3/14	21	13/57	23	2/6	33	14/65	22
Retinal hyporeflective space	4/71	6	1/14	7	3/57	5	1/6	17	3/65	5
Intraretinal fluid	1/68	1	0/15	0	1/53	2	0/8	0	1/60	2
Large outer retinal cystic spaces	7/73	10	2/14	14	5/59	8	0/7	0	7/66	19
Subretinal fluid	1/68	1	0/15	0	1/53	2	1/8	13	0/60	0
RPE thickening / bumps	14/26	54	2/3	67	12/23	52	2/3	67	12/23	52
RPE atrophy	9/26	35	1/3	33	8/23	35	1/3	33	8/23	35
Bowing of retina-RPE-Bruch's membrane	6/73	8	0/14	0	6/59	10	1/7	14	5/66	8
Choroidal hyporeflectivity / shadowing	25/41	61	5/8	63	20/33	61	2/4	50	23/37	62
Choroidal hyperreflectivity	7/41	17	1/8	13	6/33	18	1/4	25	6/37	16
Choroidal thickening	22/40	55	3/6	50	19/34	56	2/5	40	20/35	57

ADJACENT TO LESION	N	%	N	%	N	%	N	%	N	%
Disorganized retinal layers	8/73	11	2/14	14	6/59	10	0/7	0	8/66	12
Subretinal or intraretinal fluid	11/73	15	1/14	7	10/59	17	1/7	14	10/66	15
Hyperreflective intraretinal dots	9/73	12	2/14	14	7/59	12	1/7	14	6/66	9
Choroidal perivascular hyperreflective dots	4/73	5	2/14	14	2/59	3	1/7	14	3/66	5
VITREOUS	N	%	N	%	N	%	N	%	N	%
Normal appearance	16/80	20	6/15	40	10/65	15	0/8	0	16/72	35
Hyperreflective dots	64/80	80	9/15	60	55/65	85	8/8	100	56/72	78
Vitreoschisis	2/80	3	1/15	7	1/65	2	1/8	13	1/72	1
Hyperreflective deposits $\geq$ 25 µm	29/80	36	6/15	40	23/65	35	5/8	63	24/72	33
Posterior hyaloid thickening over lesion	28/80	35	3/15	20	25/65	38	3/8	38	25/72	35
Posterior hyaloid attached at lesion	70/80	88	14/15	93	56/65	86	6/8	75	64/72	89
Traction adjacent to lesion	1/80	1	0/15	0	1/65	2	0/8	0	1/72	1
Partial posterior vitreous detachment	13/80	16	2/15	13	11/65	17	1/8	13	12/72	17
Epiretinal membrane at lesion	4/80	5	1/15	7	3/65	5	0/8	0	4/72	6

Abbreviations: SD-OCT = spectral domain optical coherence tomography; TRC toxoplasmic retinochoroiditis; IgM = immunoglobulin M; HIV = human immunodeficiency virus; RPE = retinal pigment epithelial/epithelium.

Supplementary Table S2. Posterior segment features (including macular signs) on SD-OCT that were observed during follow-up of active TRC in 15 eyes of 15 patients (SD-OCT of the lesion) or 23 eyes of 23 patients (SD-OCT of the macula), who were followed for at least 8 weeks and did not have a recurrence.

**SD-OCT feature at follow-up**		
LESION	N	%
Hyaloid thickening	8/15	53
ERM at lesion	4/15	27
Expanded posterior vitreous detachment	3/15	20
Intra or subretinal fluid	0/15	0
Disorganized retinal layers	11/15	73
Retinal hyperreflectivity	3/15	20
Retinal hyporeflective space	2/15	13
Relatively thinned retina	6/15	40
Relatively thickened retina	2/15	13
RPE thickening	6/15	40
RPE atrophy	2/15	13
Adjacent RPE thickening	3/15	20
Adjacent RPE atrophy	2/15	13
Choroidal shadowing / hyporeflectivity	3/15	20
Choroidal hyperreflectivity	3/15	20
MACULA	N	%
ERM	10/23	43
Intraretinal fluid	0/23	0
Retinal hyperreflectivity	0/23	0
Retinal hyporeflective space	3/23	13
Subretinal fluid	3/23	13
Vitreomacular traction	1/23	4
Subretinal hyperreflectivity	1/23	4

Abbreviations: SD-OCT = spectral domain optical coherence tomography; TRC = toxoplasmic retinochoroiditis; ERM = epiretinal membrane, RPE = retinal pigment epithelial.
